# Nano-Delivery of Immunogenic Cell Death Inducers and Immune Checkpoint Blockade Agents: Single-Nanostructure Strategies for Enhancing Immunotherapy

**DOI:** 10.3390/pharmaceutics16060795

**Published:** 2024-06-12

**Authors:** Yujeong Moon, Hanhee Cho, Kwangmeyung Kim

**Affiliations:** 1 Department of Bioengineering, Korea University, Seoul 02841, Republic of Korea; phoenix0310@kist.re.kr; 2 Medicinal Materials Research Center, Biomedical Research Division, Korea Institute of Science and Technology (KIST), Seoul 02792, Republic of Korea; 3 Graduate School of Pharmaceutical Sciences, College of Pharmacy, Ewha Womans University, Seoul 03760, Republic of Korea; ricky@ewha.ac.kr

**Keywords:** cancer immunotherapy, immune checkpoint blockades, immunogenic cell death, nanocarriers, polymeric nanoparticles, liposomes, peptide-based nanoparticles, inorganic nanoparticles

## Abstract

Cancer immunotherapy has revolutionized oncology by harnessing the patient’s immune system to target and eliminate cancer cells. However, immune checkpoint blockades (ICBs) face limitations such as low response rates, particularly in immunologically ‘cold’ tumors. Enhancing tumor immunogenicity through immunogenic cell death (ICD) inducers and advanced drug delivery systems represents a promising solution. This review discusses the development and application of various nanocarriers, including polymeric nanoparticles, liposomes, peptide-based nanoparticles, and inorganic nanoparticles, designed to deliver ICD inducers and ICBs effectively. These nanocarriers improve therapeutic outcomes by converting cold tumors into hot tumors, thus enhancing immune responses and reducing systemic toxicity. By focusing on single-nanoparticle systems that co-deliver both ICD inducers and ICBs, this review highlights their potential in achieving higher drug concentrations at tumor sites, improving pharmacokinetics and pharmacodynamics, and facilitating clinical translation. Future research should aim to optimize these nanocarrier systems for better in vivo performance and clinical applications, ultimately advancing cancer immunotherapy.

## 1. Introduction

Cancer immunotherapy has emerged as a novel and innovative therapeutic method in the field of oncology in recent years [1,2]. Unlike conventional chemotherapeutic agents, immunotherapeutic agents enhance patients’ own immune systems by blocking immune checkpoint proteins on the surface of cancer cells, thereby inhibiting the immune evasion mechanisms of tumors [3,4]. Moreover, by memorizing the immunogenicity of target tumors, immunotherapy can treat tumors ranging from the localized to metastatic stages [5]. Due to these effective performances, immune checkpoint blockades (ICBs) such as programmed cell death 1 (PD-1; nivolumab, pembrolizumab, cemiplimab), programmed cell death ligand 1 (PD-L1; avelumab, durvalumab, atezolizumab), cytotoxic T-lymphocyte associated protein 4 (CTLA-4; ipilimumab), and lymphocyte-activation gene 3 (LAG-3; relatlimab) antibodies have been approved by the U.S. Food and Drug Association (FDA) and utilized in actual patients for various types of tumors [6,7,8]. However, ICBs have severe limitations—in particular, their low response rate in patients—despite their revolutionary effect in some cases [9].

The low patient response rate to ICBs is observed across various types of immunotherapies and is attributed to the ‘cold tumor’ phenotype, characterized by a lack of immunogenicity [10,11,12,13]. Unlike ‘hot tumors’, which are rich in tumor-infiltrating lymphocytes (TILs) and surface immune checkpoint proteins, cold tumors exhibit minimal amounts of TILs and these proteins [14,15]. Additionally, the tumor microenvironment of cold tumors includes features such as a stiff extracellular matrix (ECM), insufficient tumor-associated antigens (TAAs), and an overexpression of immunosuppressive cytokines [16,17,18,19]. These characteristics contribute to a failure in tumor cell recognition, the acceleration of regulatory T cell (Treg) differentiation, and the suppression of cytotoxic T cell proliferation, thereby facilitating evasion from the in vivo immune system [16,20]. Therefore, enhancing the immunogenicity of tumors is emphasized as being crucial to success in cancer immunotherapy [21].

To increase the response rate in immunotherapy by converting cold tumors to hot tumors, inducing immunogenic cell death (ICD) has emerged as the most popular strategy among various approaches [14,22,23,24,25,26]. ICD inducers, such as anthracyclines and photodynamic therapy, can modify the immunosuppressive tumor microenvironment by facilitating the release of TAAs and damage-associated molecular patterns (DAMPs), including the exposure of calreticulin (CRT) and the release of high-mobility group box 1 (HMGB1) and adenosine triphosphate (ATP) [27,28,29]. These immune-modulatory signals attract dendritic cells (DCs), resulting in enhanced tumor antigen presentation, which in turn increases cytotoxic T cell activity through a synergistic effect that enhances T cell infiltration [30,31,32,33,34,35]. Through these mechanisms, ICD can successfully break the tolerance and immune suppression characteristic of cold tumors, shifting the immune response towards a T helper cell type 1 (Th1-type), which is crucial in the eradication of tumor cells and the establishment of long-term immunological memory [27,36,37]. However, the use of potent cytotoxic agents in ICD is associated with severe side effects, presenting challenges in the conversion of tumor types [38,39,40,41,42,43].

To overcome the limitations mentioned above, employing a nanocarrier for drug delivery systems could be the optimal choice [10,44,45]. Tumor accumulation in nanocarriers, facilitated by the enhanced permeation and retention (EPR) effect, is well-documented and helps in reducing off-target toxicity [46,47,48,49]. Numerous reports have detailed several drug delivery systems utilizing polymers, inorganic materials, lipids, and self-assembled nanoparticles, all capable of releasing encapsulated cytotoxic agents directly into tumor tissues [10,45,50,51,52]. Utilizing these advanced drug delivery systems enables a more controlled and targeted approach, minimizing the systemic dispersion of cytotoxic agents and thereby significantly reducing the adverse side effects associated with ICD inducers [39]. Furthermore, by achieving higher concentrations of therapeutic agents at the tumor site, these systems not only reduce the likelihood of side effects but also enhance the overall efficacy of immunotherapy, promoting a more potent and specific immune response against the tumor [53,54].

**Figure 1:**
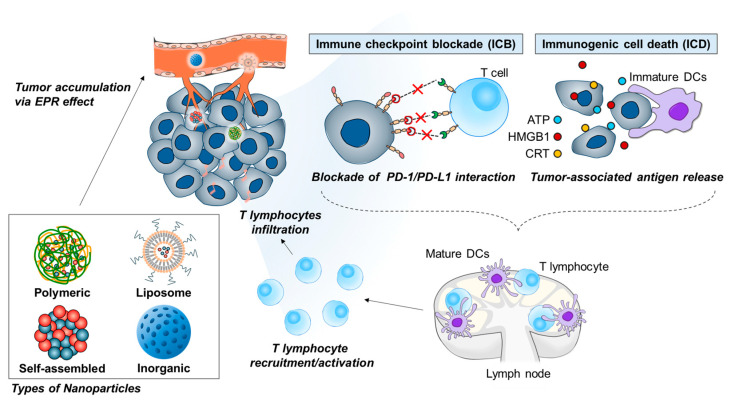
Schematic illustration of the action mechanism of nanoparticles encapsulating ICD and ICB agents in cancer immunotherapy.

Herein, we aim to introduce a categorized nano-delivery system capable of delivering ICD inducers and ICBs to enhance immunotherapy by converting cold tumors into hot tumors (Figure 1). Specifically, we focus on studies utilizing a single nanoparticle that can simultaneously deliver both ICD inducers and ICBs, thereby preventing the suppression of synergistic effects due to differing delivery efficiencies. Furthermore, the use of a single-nanoparticle-based drug delivery system facilitates the evaluation of the pharmacokinetics and pharmacodynamics of co-encapsulated ICD inducers and ICBs (Table 1).

## 2. Polymeric Nanoparticles for ICD/ICB Combination Therapy

Polymer nanoparticles are synthesized from both natural and synthetic polymers, offering a versatile and controlled delivery platform that is highly effective in cancer therapy [64]. Their biodegradability and biocompatibility are critical in minimizing toxicity, an essential aspect in oncological applications, while facilitating the delivery of a diverse array of therapeutics, ranging from chemotherapeutic agents to genetic materials [65]. The modification of surface properties in these nanoparticles enhances circulation times and enables the precise targeting of tumor cells, which is pivotal in reducing off-target effects and enhancing therapeutic efficacy [66]. Additionally, polymer nanoparticles can be engineered to incorporate responsive design features that trigger drug release in reaction to specific conditions within the tumor microenvironment, such as an acidic pH or elevated enzyme concentrations, ensuring localized drug release at the tumor site [67,68]. For instance, PLGA (poly(lactic-co-glycolic acid)) nanoparticles are extensively utilized in clinical settings due to their excellent safety profile and effective drug release properties, rendering them ideal for sustained and controlled drug delivery in oncological treatments [69]. Due to these properties of polymeric nanoparticles, various block copolymers have been developed and utilized for the fabrication of polymeric nanoparticles, and research on their fabrication methods has been actively conducted [70].

**Figure 2:**
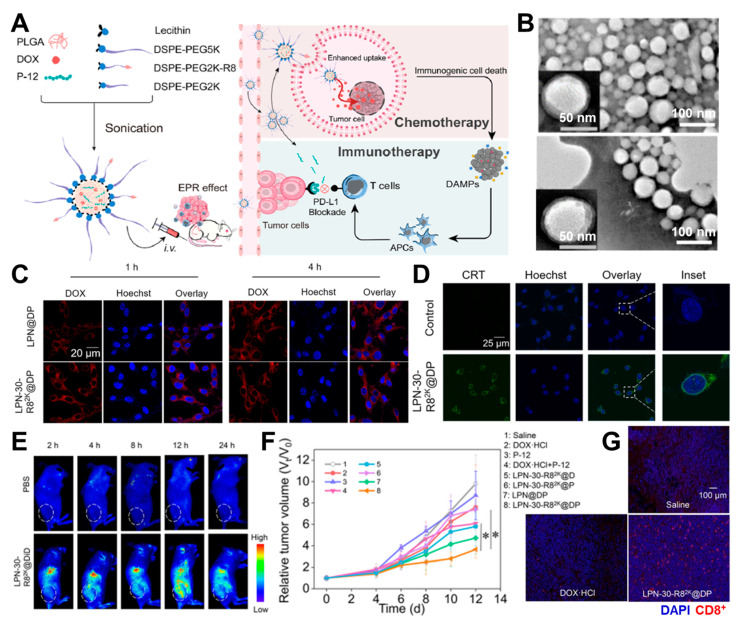
(**A**) Preparation of DOX/P-12-encapsulated cell-penetration peptide octaarginine (R8) conjugated lipid/PLGA nanoparticles and their mechanism of action. (**B**) TEM images of LPN (**top**) and LPN-30-R8^2k^ (**bottom**) with hydrodynamic volumes of 100 nm and 125 nm, respectively. (**C**) Enhanced cellular uptake of LPN-30-R8^2k^@DP in CT26 cells compared to LPN@DP. (**D**) Expression of CRT as DAMPs due to DOX release from LPN-30-R8^2k^@DP. (**E**) Improved tumor targeting efficiency of LPN-30-R8^2k^@DP via passive targeting in CT26-tumor bearing mice. The white dashed line indicates the location of the tumor. (**F**) Enhanced tumor growth suppression using LPN-30-R8^2k^@DP compared to other groups with a dosage of 3 mg dox/kg and 5 mg P-12/kg. (**G**) CD8^+^ T cell staining in excised tumor tissue from the LPN-30-R8^2k^@DP treated group. The student’s *t*-test was applied for statistical significance. (* *p* < 0.05). Reproduced with permission [55]. Copyright: ACS publications, 2022.

Zhang et al. developed lipid/PLGA nanocomplexes (LPNs) for combination therapy using DOX and PD-L1 blockade [55]. They encapsulated DOX and the anti-PD-L1 peptide P-12 within PLGA and 1,2-distearoyl-sn-glycero-3-phosphoethanolamine-N-(polyethylene glycol) (DSPE-PEG) nanocomplexes, which were further surface-modified with a cell-penetrating peptide, octaarginine (LPN-30-R8^2k^@DP) (Figure 2A). Despite the DSPE-PEG-R8 surface modification, the nanocomplexes maintained a spherical structure, without significant size changes (Figure 2B). The inclusion of R8 peptides on LPN-30-R8^2k^@DP enhanced cellular uptake compared to LPN@DP, which lacked the R8 peptide (Figure 2C). Subsequently, DOX released from LPN-30-R8^2k^@DP induced DAMPs with calreticulin (CRT) expression on target cells (Figure 2D). These nanocomplexes effectively delivered to the tumor site via the EPR effect (Figure 2E) and demonstrated significant tumor suppression, accompanied by the proliferation of CD8^+^ cytotoxic T cells mediated by the LPN-loaded P-12 peptide (Figure 2F,G).

Song et al. developed an all-in-one platform based on glycol chitosan nanoparticles (CNPs) to deliver both DOX and an anti-PD-L1 peptide [56]. They chemically conjugated the _D_-form peptide PP (NYSKPTDRQYHF) to CNPs, creating stable PP-CNPs capable of effective multivalent binding with PD-L1 proteins on tumor cell surfaces. This binding facilitated the lysosomal degradation of PD-L1, effectively preventing its recycling, a common issue with traditional anti-PD-L1 antibodies. Consequently, PP-CNPs downregulated PD-L1, inhibiting tumor immune evasion in CT26 colon cancer mouse models. Additionally, by loading DOX into PP-CNPs, they created DOX-PP-CNPs, which induced ICD. This combination therapy demonstrated enhanced tumor targeting through both passive and active mechanisms, leading to effective tumor-specific ICD without toxicity toward normal cells. This approach resulted in the significant downregulation of tumor PD-L1 proteins, achieving a high rate of complete tumor regression (60%) in CT26-tumor bearing mice.

Dual drug-loaded nanoparticles were developed by Phung and colleagues to deliver low-dose DOX to induce ICD and miR-200c for PD-L1 inhibition [57]. They utilized two block copolymers, folic acid (FA)-conjugated PLGA-PEG (PLGA-PEG-FA) and PLGA-polyethyleneimine (PLGA-PEI), to form folate-targeted nanoparticles. These nanoparticles had a size of 110.4 ± 2.1 nm, with a narrow size distribution of 0.19 ± 0.02, ensuring serum stability. FA modification enhanced the nanoparticle uptake by cancer cells in vitro and increased accumulation in tumor microenvironments in vivo. The loaded drugs induced ICD and inhibited PD-L1, leading to improved dendritic cell maturation and CD8^+^ T cell activation both in vitro and in vivo. This resulted in significant tumor growth inhibition, unlike treatments with free DOX, miR-NPs, or DOX-NPs.

## 3. Liposomes for ICD/ICB Combination Therapy

Liposomes are exceptionally beneficial in cancer therapy due to their biocompatibility and their ability to emulate cell membranes, which diminishes immune clearance and enhances the efficiency of drug delivery [58]. Their structural versatility facilitates the encapsulation of both hydrophilic and lipophilic drugs, providing a substantial advantage in administering a range of anticancer drugs with varying solubilities [23,60]. Liposomes effectively utilize the EPR effect for passive targeting to tumor sites, where they preferentially accumulate compared to normal tissues, thus increasing the local drug concentration at the tumor site while minimizing systemic side effects [71]. Furthermore, sophisticated liposome formulations may include surface modifications with antibodies or ligands that actively target cancer cells, significantly enhancing the specificity and effectiveness of the therapy [24,60,72]. The clinical efficacy of liposomal formulations, exemplified by Doxil (liposomal doxorubicin), underscores their role in reducing toxicity and improving the therapeutic index of traditional cancer treatments [73]. Furthermore, liposomes with various appropriate characteristics are currently being fabricated via alterations in their building blocks, and there are active studies on fabrication methods aimed at achieving more uniform and precise quality control [74,75].

**Figure 3:**
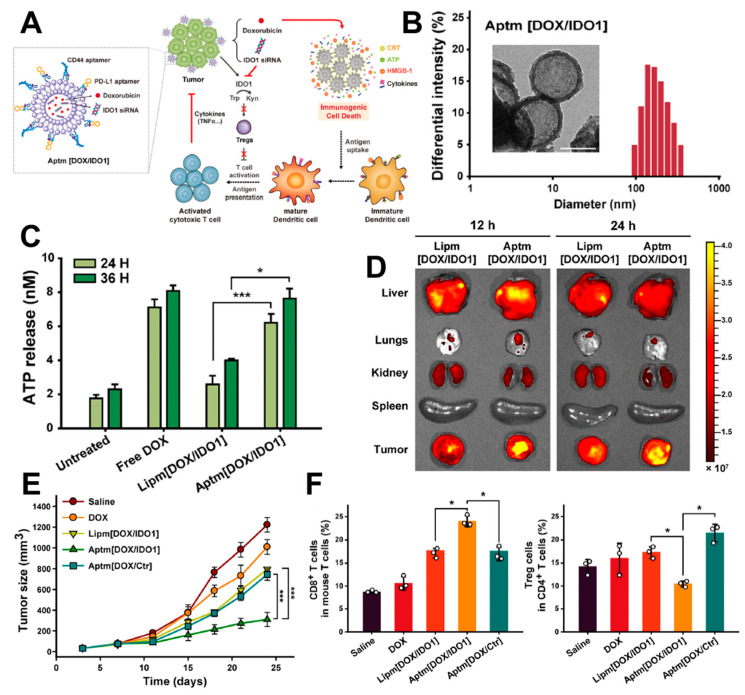
(**A**) Preparation of DOX/IDO1-encapsulated liposomes surface-modified with CD44 and PD-L1 aptamers, and their mode of action. (**B**) The size of the nanostructures confirmed by DLS and TEM images with an average size of 183 nm. (**C**) Released DOX from the liposomes induced ATP release as DAMPs in MDA-MB-231 cells. (**D**) Aptm[DOX/IDO1] demonstrated enhanced tumor targeting in 4T1-tumor-bearing mice compared to Lipm[DOX/IDO1], due to a dual-targeting mechanism via passive and active targeting. (**E**) Effective tumor suppression was observed in the Aptm[DOX/IDO1]-treated group in the 4T1 tumor xenograft model. (**F**) Immune activation was evident in the excised tumor tissue from the Aptm[DOX/IDO1]-treated group. One-way analysis of variance (ANOVA) with Dunnett’s test was applied for statistical significance (* *p* < 0.05, *** *p* < 0.005). Reproduced with permission [58]. Copyright: Elsevier, 2022.

For the effective delivery of DOX and PD-L1 blockade, Kim et al. introduced a liposome-based delivery system for DOX and indoleamine 2,3-dioxygenase 1 (IDO1), incorporating surface modifications with CD44 and PD-L1 aptamers (Aptm[DOX/IDO1])(Figure 3A) [58]. This multifunctional liposome displayed a spherical nanostructure with an average size of 183 nm, optimized for effective passive tumor targeting (Figure 3B). In cell treatment studies, they observed an upregulation of DAMPs, specifically ATP release induced by the DOX released from the encapsulated liposomes (Figure 3C). The Aptm[DOX/IDO1] liposomes demonstrated superior tumor targeting efficiency compared to Lipm[DOX/IDO1], which lacked surface modifications (Figure 3D). This enhancement was attributed to the dual-targeting capability of Aptm[DOX/IDO1], achieving both passive and active targeting through the aptamers, whereas Lipm[DOX/IDO1] relied solely on passive targeting. Consequently, Aptm[DOX/IDO1] exhibited the most effective tumor growth suppression (Figure 3E), alongside increased CD8^+^ T cell activity and the downregulation of regulatory T cells, underscoring its potential in improving cancer immunotherapy outcomes (Figure 3F).

Wang et al. developed a novel reactive oxygen species (ROS)-activated liposome nanoplatform designed to address the challenges of paclitaxel delivery, such as premature drug release and low tumor accumulation [59]. They loaded an ROS-sensitive paclitaxel derivative (PSN) into liposomes to enhance drug stability and targeting. The liposomal nanosystem was innovatively designed to use the remote loading of BMS-202, a small molecule PD-1/PD-L1 inhibitor, alongside PSN for ROS-sensitive paclitaxel release and sustained BMS-202 release. This co-loading strategy resulted in a high co-loading efficiency and improved pharmacokinetic properties. The efficacy of this nanoplatform was evaluated using an orthotopic 4T1 breast cancer model, demonstrating superior antitumor activity. Paclitaxel-mediated ICD activated antitumor immunity, while the continuous blockade of PD-L1 by BMS-202, which could be upregulated by paclitaxel means in tumors, enhanced the response to ICB and restored host immune surveillance.

The rational design of PD-L1 multivalent binding liposomes was investigated by Yang and colleagues through the incorporation of varying ratios of PD-L1 binding peptides into PEGylated liposomes [60]. They discovered that liposomes with 10 mol% PD-L1 binding peptides (10-PD-L1-Lipo) promoted effective multivalent binding with PD-L1 on tumor cell surfaces. This binding facilitated endocytosis and trafficking to lysosomes, significantly degrading PD-L1 and reducing its cellular abundance compared to anti-PD-L1 antibodies. Consequently, 10-PD-L1-Lipo disrupted tumor immune escape mechanisms and enhanced T cell-mediated antitumor immunity. Furthermore, they developed a DOX liposomal formulation, DOX-PD-L1-Lipo, which primed tumors through immunogenic chemotherapy via preferential DOX accumulation and overcame chemotherapy-induced PD-L1 upregulation, resulting in significantly enhanced antitumor efficacy and immune responses in colon tumor models.

## 4. Peptide-Based and Inorganic Nanoparticles for ICD/ICB Combination Therapy

In addition to the nanostructures previously described, there are various methods for forming diverse types of nanostructures [10,76]. For example, amphiphilic peptide-based self-assembling nanoparticles do not require separate carriers, leading to a high drug encapsulation efficiency and eliminating carrier-related toxicity [77,78,79,80]. Another category includes inorganic nanoparticles such as gold, silver, and silica [81,82,83]. These nanoparticles offer unique advantages in cancer therapy due to their distinctive physical properties [84]. Specifically, they can be engineered to absorb light and generate heat, making them ideal candidates for photothermal therapy—a targeted technique designed to directly eradicate tumor cells [85,86].

**Figure 4:**
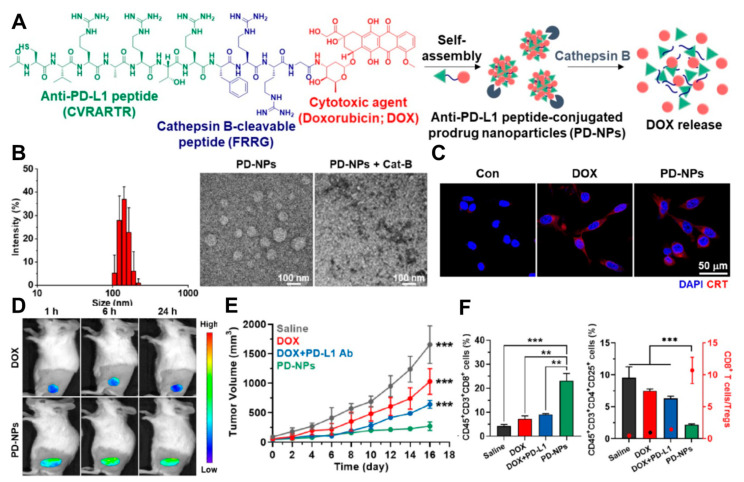
(**A**) Preparation of self-assembled peptide-derived prodrug nanoparticles capable of releasing DOX in the presence of cathepsin B. (**B**) The size of the self-assembled nanoparticles was confirmed by DLS and TEM with an average size of 157.4 ± 12.1 nm, while no particle formation was observed in the presence of cathepsin B. (**C**) CRT expression was observed as DAMPs due to the specific release of DOX in 4T1 cancer cells. (**D**) Enhanced tumor-targeting efficiency of nanoparticles via the EPR effect in the 4T1 tumor xenograft model at 150–200 mm^3^ volume. (**E**) Improved tumor suppression due to the release of DOX and PD-L1 blockade peptide with a dosage of 3 mg DOX/kg. (**F**) Immune analysis (CD8^+^ cytotoxic T cell, regulatory T cell) of excised tumor tissue following tumor suppression evaluation. Reproduced with permission [61]. One-way analysis of variance (ANOVA) with Tukey-Kramer *posthoc* test was applied for statistical significance (** *p* < 0.01, *** *p* < 0.005). Copyright: Ivyspring international Publisher, 2022.

Moon et al. are among the researchers utilizing peptide-derived self-assembled nanoparticles for ICD/ICB combination cancer therapy [61]. They first designed peptide–drug conjugates (PD-NPs) composed of an anti-PD-L1 peptide (CVRARTR), a cathepsin B cleavable linker (FRRG), and DOX, which could self-assemble into cathepsin B-degradable nanoparticles (Figure 4A). They demonstrated the nanoformulation of PD-NPs in an aqueous solution, which degraded upon the addition of cathepsin B enzyme (Figure 4B). Due to tumor-specific DOX release, PD-NPs induced CRT expression as DAMPs, facilitating the transformation of cold tumors into hot tumors (Figure 4C). The PD-NPs accumulated effectively at the tumor site via passive targeting (Figure 4D), leading to enhanced tumor growth suppression through the cancer-specific release of DOX and anti-PD-L1 peptide (Figure 4E). This effect was further demonstrated through the upregulation of CD8^+^ T cells and the downregulation of CD25^+^ regulatory T cells, resulting in the highest T cell/Treg ratio for PD-NPs compared to other groups (Figure 4F).

Emami et al. developed a combination therapy using DOX-conjugated and anti-PD-L1-targeting gold nanoparticles (PD-L1-AuNP-DOX) for targeted chemo-photothermal therapy for colorectal cancer (CRC) [62]. DOX and anti-PD-L1 antibodies were conjugated to the α-terminal end of lipoic acid polyethylene glycol N-hydroxysuccinimide (LA-PEG-NHS) using an amide linkage. The nanoparticles (40.0 ± 3.1 nm) were constructed by linking LA-PEG-DOX, LA-PEG-PD-L1, and a short PEG chain to gold nanoparticles (AuNPs) via thiol–Au covalent bonds. Characterization and biological studies under near-infrared (NIR) irradiation showed an efficient intracellular uptake of DOX, evidenced by pronounced apoptotic effects (66.0%) in CT-26 cells. Combined PD-L1-AuNP-DOX and NIR irradiation treatment significantly suppressed the proliferation of CT-26 cells by increasing apoptosis and inducing cell cycle arrest.

To improve the immunosuppressive microenvironment of difficult-to-treat tumors and enhance immunotherapy efficacy, Wang et al. developed an advanced mesoporous nanocarrier [63]. This nanocarrier features an upconverting nanoparticle core with a large-pore mesoporous silica shell (UCMS) loaded with photosensitizer molecules, the IDO-derived peptide vaccine AL-9, and a PD-L1 inhibitor. The IDO-derived peptide targets IDO-expressing tumor cells by being recognized by dendritic cells and presented to CD8+ cytotoxic T cells. Simultaneously, near-infrared (NIR)-activated photodynamic therapy (PDT) induces immunogenic cell death (ICD), promoting effector T cell infiltration. This integrated approach combining PDT-induced ICD, peptide vaccination, and immune checkpoint blockade significantly boosts both local and systemic antitumor immunity, demonstrating a synergistic strategy for enhancing cancer immunotherapy.

## 5. Conclusions

The utilization of nanocarrier systems in cancer immunotherapy has shown significant promise in enhancing the efficacy of treatments through the targeted delivery of ICD inducers and ICBs. These systems, including polymeric nanoparticles, liposomes, peptide-based nanoparticles, and inorganic nanoparticles, offer several advantages, such as improved biocompatibility, precise targeting, and the ability to co-deliver multiple therapeutic agents. By facilitating the conversion of cold tumors to hot tumors, these nanocarriers enhance immune responses and overcome the limitations associated with traditional immunotherapy, such as low patient response rates and systemic toxicity. Future research should focus on optimizing these nanocarrier designs, understanding their in vivo behavior, and translating these advanced systems into clinical practice to improve cancer treatment outcomes.

## Figures and Tables

**Table 1:** Categorized nanoparticles for the effective delivery of ICD and ICB agents.

Category	Type	ICD	ICB	Ref.
Polymeric	PLGA/lipid	Doxorubicin (DOX)	Anti-PD-L1 peptide(P-12)	[55]
	Chitosan Nanoparticle	DOX	Anti-PD-L1 peptide(NYSKPTDRQYHF)	[56]
	PLGA-PEG-FA PLGA-PEI	DOX	miRNA-200c	[57]
Liposome	PEGylated	DOX	PD-L1 aptamer	[58]
	PEGylated	Paclitaxel	BMS-202	[59]
	PEGylated	DOX	Anti-PD-L1 peptide(NYSKPTDRQYHF)	[60]
Peptide	Self-assembled	DOX	Anti-PD-L1 peptide(CVRARTR)	[61]
Inorganic	Gold	DOX	PD-L1 antibody	[62]
	Mesoporous silica	Rose bengal (PDT)	PD-L1 antibody	[63]
